# Treatment of Statin-Induced Necrotizing Autoimmune Myopathy With Glucocorticoid Monotherapy

**DOI:** 10.7759/cureus.12086

**Published:** 2020-12-14

**Authors:** Matthew Lempel, Ermal Molla

**Affiliations:** 1 Internal Medicine, Waterbury Hospital, Waterbury, USA; 2 Rheumatology, Waterbury Hospital, Waterbury, USA

**Keywords:** statin-induced necrotizing autoimmune myopathy, statin-associated autoimmune myopathy, immune-mediated necrotizing myopathy

## Abstract

Statins are widely prescribed medications to prevent cardiovascular events such as myocardial infarction and stroke. Both myalgia and myopathy are well-known potential side effects of statins. However, a rare and severe form of statin-induced necrotizing autoimmune myopathy (SINAM) has recently been described and can lead to debilitating weakness, often requiring immunosuppressive therapy. We report a case of a 73-year-old male who made a complete recovery from SINAM following a three-month course of prednisone monotherapy.

## Introduction

Myalgia related to statin administration is generally a self-limited side effect. Nevertheless, it is now recognized that a more severe autoimmune myopathy characterized by muscle necrosis can develop in approximately 0.002% of patients treated with statins [1]. As this is an uncommon phenomenon, recommendations to help guide evidence-based treatment are limited.

## Case presentation

**Figure 1:**
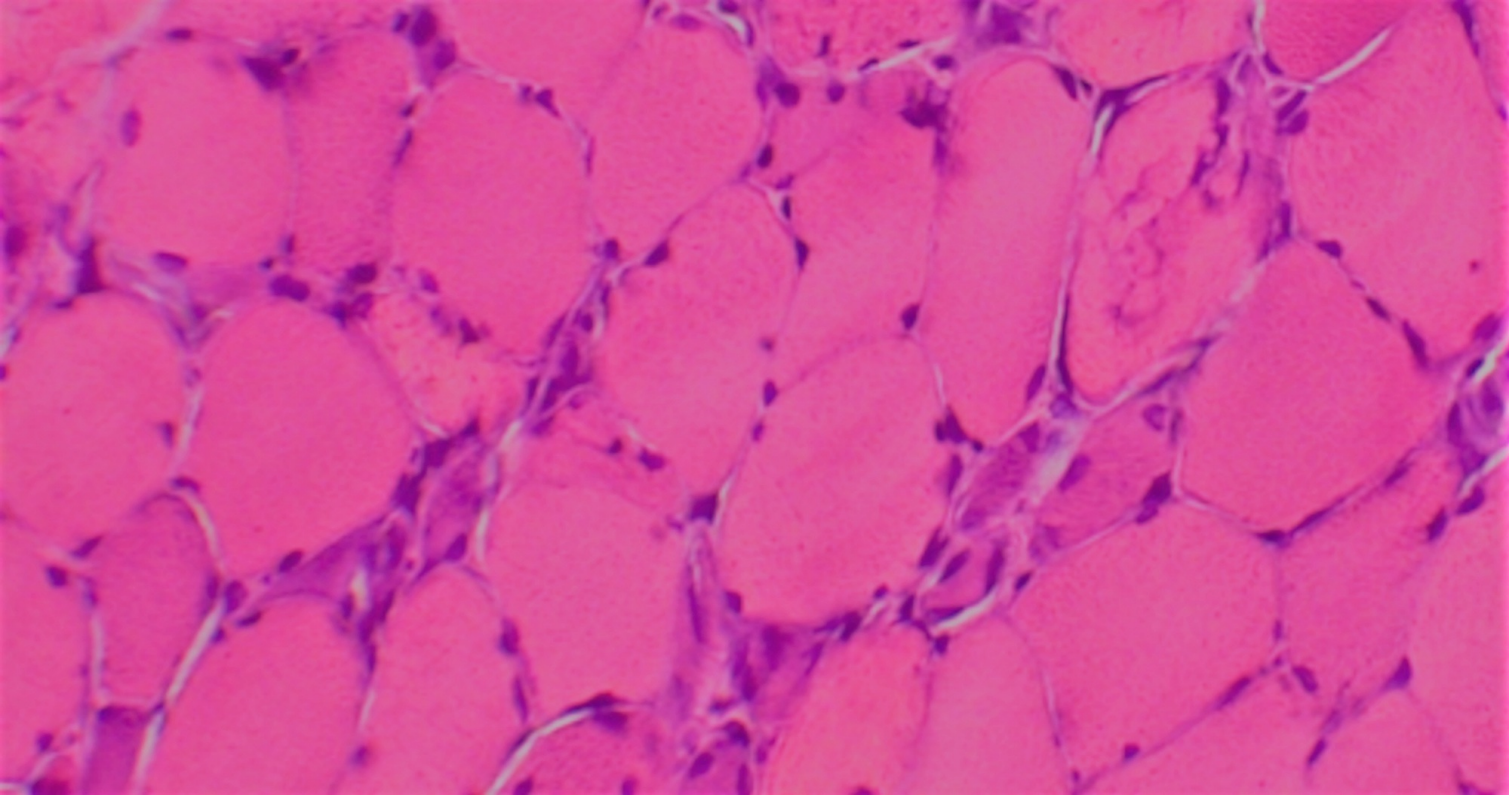
Muscle biopsy Hematoxylin-eosin–stained section of quadriceps muscle showing numerous necrotic fibers.

A 73-year-old male with a past medical history of complete heart block status post dual-chamber pacemaker insertion and hypertension presented to his primary care physician due to progressive generalized weakness of two-months duration. Physical exam was significant for 3/5 symmetric proximal strength, and initial laboratory workup demonstrated a creatine kinase (CK) level of 8,915 IntUnit/L with an associated mild transaminitis. As the patient had been on atorvastatin 40 mg daily for several years, a preliminary diagnosis of rhabdomyolysis from statin-associated myopathy was presumed, and inpatient admission for intravenous fluid administration was recommended. Following a mild decrease in the CK level, the patient was discharged off statin therapy with recommended outpatient follow up. Due to persistent objective weakness despite the cessation of statin therapy, CK and aldolase levels were rechecked two months later and found to be 1,600 IntUnit/L and 52.2, respectively. After a myositis panel came back benign, an anti-3-hydroxy-3-methyl-glutaryl-coenzyme A (anti-HMG-CoA) reductase antibody was ordered and returned positive at 118 units (normal <20 units). As seen in Figure 1, an ensuing biopsy of the left quadriceps muscle demonstrated “scattered myofiber necrosis and regeneration without inflammation.” Following the confirmation of statin-induced necrotizing autoimmune myopathy (SINAM), prednisone 60 mg daily was introduced. As the patient continued to improve, the corticosteroids were quickly tapered over ninety-days without any significant adverse effects. Despite remaining off any immunosuppressant for six months, the patient has returned to his original degree of strength and function.

## Discussion

While the primary mechanism behind SINAM remains unknown, statin exposure does lead to increased expression of HMG-CoA reductase. Moreover, binding of statin to 3-hydroxy-3-methyl-glutaryl-coenzyme A (HMG-CoA) reductase can change the confirmation of the protein, which in theory could affect immune system tolerance. Once tolerance is broken, high HMG-CoA reductase levels in regenerating muscle could continue to drive autoimmunity even after statin therapy is discontinued [1].

As the development of an autoimmune myopathy secondary to statin exposure is exceedingly rare, no randomized trials or large enough case series are available on which to base definitive treatment recommendations. However, in a recently published meta-analysis, Nazir et al. found that 84% of reported SINAM cases required two or more immunosuppressants [2]. Alternatively, several cases have described spontaneous improvement with discontinuation of statin therapy alone [3-4]. While no formal treatment guideline exists, the 224th European Neuromuscular Centre outlines several possible treatment approaches starting with high dose prednisone or pulsed intravenous methylprednisolone as first line agents [5]. If a prolonged course of corticosteroid is anticipated or if the dose cannot be lowered to less than 10 mg/day, one can add azathioprine at a dose of 3 mg/kg or methotrexate at a dose of 20-25 mg per week. As both possess hepatotoxicity, mycophenolate mofetil can be introduced at 2-3 grams daily in divided doses. Drugs of last resort include rituximab, cyclosporine, cyclophosphamide and etanercept [5]. Alternatively, cessation of disease progression and improvement in overall strength and CK level is well described with intravenous immunoglobulin administration [6].

## Conclusions

Although rare, SINAM should be considered in patients with exposure to a statin, presenting with a persistent myopathy, particularly if the offending agent has already been discontinued. Unlike statin-induced myopathy, individuals with SINAM should never be rechallenged with a statin. While this patient made a complete recovery without adding steroid-sparing agents or a second immunosuppressant, large randomized controlled trials comparing different therapeutic regimens are needed to help guide clinical management.
